# Computational Identification of Potential Anti-Inflammatory Natural Compounds Targeting the p38 Mitogen-Activated Protein Kinase (MAPK): Implications for COVID-19-Induced Cytokine Storm

**DOI:** 10.3390/biom11050653

**Published:** 2021-04-29

**Authors:** Seth O. Asiedu, Samuel K. Kwofie, Emmanuel Broni, Michael D. Wilson

**Affiliations:** 1 Department of Parasitology, Noguchi Memorial Institute for Medical Research, College of Health Sciences, University of Ghana, Legon, Accra P.O. Box LG 581, Ghana; soasiedu@noguchi.ug.edu.gh (S.O.A); MWilson@noguchi.ug.edu.gh (M.D.W); 2 Department of Biomedical Engineering, School of Engineering Sciences, College of Basic and Applied Sciences, University of Ghana, Legon, Accra P.O. Box LG 77, Ghana; ebroni002@st.ug.edu.gh; 3 West African Centre for Cell Biology of Infectious Pathogens, Department of Biochemistry, Cell and Molecular Biology, College of Basic and Applied Sciences, University of Ghana, Accra P.O. Box LG 54, Ghana; 4 Department of Medicine, Loyola University Medical Center, Maywood, IL 60153, USA

**Keywords:** COVID-19, coronavirus, p38 MAPK, cytokine storm, anti-inflammatory compounds, natural products, molecular dynamics simulation, molecular docking

## Abstract

Severely ill coronavirus disease 2019 (COVID-19) patients show elevated concentrations of pro-inflammatory cytokines, a situation commonly known as a cytokine storm. The p38 MAPK receptor is considered a plausible therapeutic target because of its involvement in the platelet activation processes leading to inflammation. This study aimed to identify potential natural product-derived inhibitory molecules against the p38α MAPK receptor to mitigate the eliciting of pro-inflammatory cytokines using computational techniques. The 3D X-ray structure of the receptor with PDB ID 3ZS5 was energy minimized using GROMACS and used for molecular docking via AutoDock Vina. The molecular docking was validated with an acceptable area under the curve (AUC) of 0.704, which was computed from the receiver operating characteristic (ROC) curve. A compendium of 38,271 natural products originating from Africa and China together with eleven known p38 MAPK inhibitors were screened against the receptor. Four potential lead compounds ZINC1691180, ZINC5519433, ZINC4520996 and ZINC5733756 were identified. The compounds formed strong intermolecular bonds with critical residues Val38, Ala51, Lys53, Thr106, Leu108, Met109 and Phe169. Additionally, they exhibited appreciably low binding energies which were corroborated via molecular mechanics Poisson–Boltzmann surface area (MM-PBSA) calculations. The compounds were also predicted to have plausible pharmacological profiles with insignificant toxicity. The molecules were also predicted to be anti-inflammatory, kinase inhibitors, antiviral, platelet aggregation inhibitors, and immunosuppressive, with probable activity (Pa) greater than probable inactivity (Pi). ZINC5733756 is structurally similar to estradiol with a Tanimoto coefficient value of 0.73, which exhibits anti-inflammatory activity by targeting the activation of Nrf2. Similarly, ZINC1691180 has been reported to elicit anti-inflammatory activity in vitro. The compounds may serve as scaffolds for the design of potential biotherapeutic molecules against the cytokine storm associated with COVID-19.

## 1. Introduction

Coronavirus disease 2019 (COVID-19) is caused by severe acute respiratory syndrome coronavirus 2 (SARS-CoV-2) [1,2]. It is a positive-sense single-stranded RNA virus [3] belonging to the *Coronaviridae* family. They are uniquely enveloped non-segmented viruses with large surface spike proteins observed as corona (crown-like) projections using electron microscopy [4]. The virus is transmitted through droplets, contacts, fecal-oral, blood, mother-to-child, airborne, fomite, and animals to humans [5,6,7,8]. COVID-19 symptoms include fever, dry cough, runny nose, sore throat, loss of taste and smell, and difficulty in breathing [9]. During critical stages, complications observed include acute respiratory distress syndrome (ARDS), pneumonia, septic shock, arrhythmia, and disseminated intravascular coagulation [9,10,11].

Several studies on these complications have reported elevated levels of plasma cytokines [12], a condition referred to as cytokine storm [13]. The p38 mitogen-activated protein kinase (MAPK) is critical in COVID-19 cytokine storms [14,15]. In the human host, angiotensin II (AngII), a pro-inflammatory peptide hormone, mediates its effects through p38 MAPK activation [14,16]. Angiotensin-converting enzyme 2 (ACE2) converts (Ang II) into angiotensin 1–7 (Ang 1–7), which binds to the Mas receptor. This counterbalances the pro-inflammatory effects of Ang II by decreasing the activation of p38 MAPK [17]. Upon cell entry, SARS-CoV-2 binds and downregulates ACE2 [18,19]. The loss of ACE2 activity upon viral entry allows for unbridled inflammation [14]. The p38 MAPK is implicated in the propagation of the SARS-CoV-2 lifecycle [14] and this results from enhanced replication due to increased MAPK activity [15]. Moreover, SARS-CoV, a close neighbor of the SARS-CoV-2 virus, expresses a protein that directly upregulates p38 MAPK in vitro [20] and this process seems to be a pathogenic step in the lifecycle of many RNA respiratory viruses [21]. A study of the effect of p38 MAPK inhibitors on SARS-CoV infected mice reported an 80% increase in survival after treatment [22]. The p38 MAPK is a plausible anti-inflammatory drug target for COVID-19 patients and its inhibitors have been trialed for the treatment of other ailments [23,24,25].

Anti-inflammatory compounds including dexamethasone are used as treatment options in COVID-19 patients [26], since no FDA-approved drugs specific for the treatment and prevention of COVID-19 exist. There are over 2300 clinical trials per the Global Coronavirus COVID-19 Clinical Trial Tracker (https://www.covid-trials.org/ (accessed on 28 September 2020)) [27] on novel vaccines, drugs, and repurposed compounds including p38 MAPK inhibitor Losmapimod.

The use of computational methods has been suggested as a viable alternative for the faster and cheaper discovery of COVID-19 therapeutic molecules [28,29,30,31,32,33]. Natural products are used as COVID-19 remedies [34,35,36,37]. This is due to their structural and chemical diversity which serves as a potent source of novel drug-like compounds [38,39]. Freely available cheminformatics-based natural compound databases such as the Traditional Chinese Database [40], African Natural Product Database [41], and the North African Natural Product Database [42] collectively contain over 30,000 unique natural products.

Consequently, the work aims at finding potential natural product-derived inhibitory molecules of p38 MAPK with the propensity to ameliorate the cytokine storm in severely ill COVID-19 patients. This study utilized computational techniques including molecular docking, dynamics simulations, and characterization of binding mechanisms using Molecular mechanics Poisson-Boltzmann surface area (MM-PBSA) calculations. It further predicted the pharmacological profiles and biological activity of drug-like and lead-like anti-inflammatory molecules as potential inhibitors of p38 MAPK.

## 2. Materials and Methods

### 2.1. Protein Structure Retrieval and Processing

The experimentally solved 3D structure of p38α MAPK was retrieved from the Protein Data Bank [43,44] with PDB ID: 3ZS5 [45]. Missing residues at 172–183 were initially added via SwissModel [46]. PyMOL v 4.0.0 [47,48] was used for preprocessing the 3ZS5 protein structure by removing water molecules and other ligands. Molecular dynamics (MD) simulation was performed using the Groningen Machine for Chemical Simulations GROMACS version 2018 [49]. Optimized Potentials for Liquid Simulations (OPLS)/All Atom (AA) force field was used to generate the protein topology and position restrain files [50,51]. Periodic Boundary Conditions (PBC) were applied to the structure with the protein centered 1 nm from the edge of a cubic box to monitor the movement of all particles and avoid edge effects on the surface atoms [52]. The system was solvated with SPC water [53,54] and neutralized with six Na atoms. The structure was energy minimized using the steepest descent algorithm at 50,000 steps.

### 2.2. Screening Library of Compounds

A library of 38,271 natural product compounds comprising 2277 compounds from the North African Natural Product Database [42], 833 compounds from the African Natural Product Database [41], and 35161 compounds from the Chinese Natural Product Database [40] was retrieved. The compounds were filtered using DataWarrior [55] with molecular weights between 250 g/mol and 350 g/mol [56,57]. Further, compounds predicted as mutagenic, causing reproductive effects, irritant, and tumorigenic were eliminated via DataWarrior. Known p38 MAPK inhibitors used as controls for the molecular docking were Losmapimod, ARRY-797, Dilmapimod, Doramapimod, Pamapimod, PH-797804, SB 202190, SB 203580, Talmapimod, VX-702, and Skepinone-L with PubChem compound IDs 11552706, 46883775, 10297982, 156422, 16220188, 22049997, 5169, 176155, 9871074, 10341154, and 45279963, respectively.

### 2.3. Validation of Docking Protocol

The easyROC [58] was used to generate the receiver operating characteristic (ROC) curve. The ROC curve was used to validate the ability of Autodock Vina to discriminate between active and decoy compounds. The SMILES of the eleven inhibitors served as inputs for the generation of 50 decoys each via Directory of Useful Decoys (DUD-E) [59]. Each decoy has similar physicochemical properties to a known inhibitor but is chemically distinct [59]. A total of 561 compounds comprising 11 actives and 550 decoys were screened against the p38 MAPK structure. The 11 actives comprised the known inhibitors of p38 MAPK.

### 2.4. Virtual Screening of Ligands

AutoDock Vina [60] was used to screen the library against the p38α MAPK protein structure. The pre-filtered library was imported into OpenBabel [61] and minimized using the Universal Force Field (Uff) for 200 steps and optimized using the conjugate gradient. A grid box of dimensions (78.35, 47.84, 49.86) Å and center (9.97, 30.65, 20.29) Å was used for docking with a default exhaustiveness of eight.

### 2.5. Pharmacological Profiling

The pharmacokinetic and physicochemical profiles of the compounds were predicted via SwissADME [62] with the Simplified Molecular Input Line-Entry System (SMILES) of the compounds as inputs.

### 2.6. Elucidation of the Protein-Ligand Interactions

LigPlot+ [63] was used to generate the 2D protein-ligand interactions with default settings. The best poses of the hits were saved in “.pdb” file formats and then visualized using PyMOL.

### 2.7. Prediction of Biological Activities of Hit Compounds

The biological activities of the hits were predicted using the Bayesian-based Prediction of Activity Spectra for Substances (PASS) [64]. The SMILES files of the compounds were used to perform structural similarity searches for antiviral and anti-inflammatory compounds with a DrugBank [65] similarity threshold of 0.7.

### 2.8. Molecular Dynamics Simulation of Protein-Ligand Complexes

MD simulations of the protein-ligand complexes were performed using GROMACS 2020 version [49]. The 3ZS5 protein topologies were generated using the GROMOS96 43a1 force field [66] in GROMACS, whereas the ligand files were generated via the PRODRG [67]. Each complex was solvated with water molecules in a cubic box of size 5.0 nm and neutralized with six Na ions. Energy minimization of each complex was conducted for 50,000 steps using the steepest descent algorithm. The ligands were restrained before the canonical ensemble, and then the isothermal-isobaric ensemble. Equilibration of each complex was performed for 100 ps apiece and final MD simulation was conducted for 100 ns with time steps of 2 fs under PME. The free binding energies were computed using G_mmpbsa [68]. The binding free energy contribution per residue was calculated using MM-PBSA and the output plots generated with R.

## 3. Results and Discussion

### 3.1. Protein Structure Retrieval and Analysis

**Figure 1:**
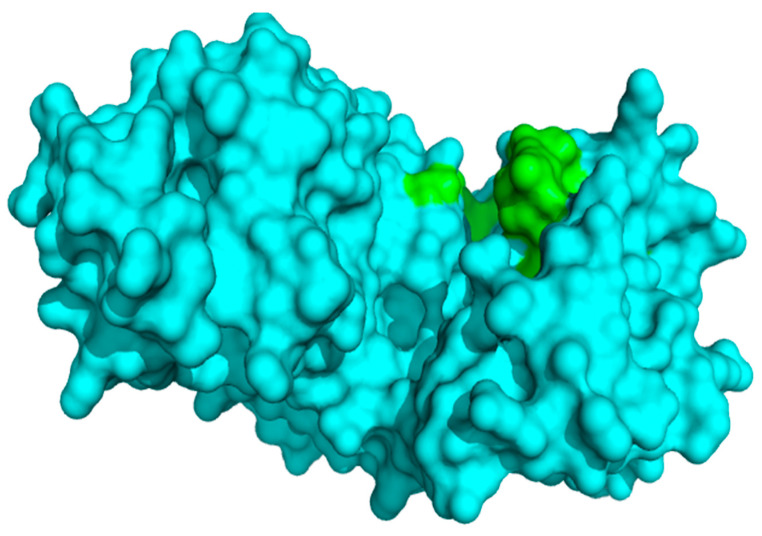
A surface representation of the structure of p38 MAPK (Chain A). The catalytic ATP binding pocket is colored green. The structure is a monomer with a binding cavity of 1365.552 Å^3^.

The PDB database contains several solved p38α MAPK structures. The p38α is the most expressed isoform of the p38 MAPK [69]. The selected protein structure with PDB ID: 3ZS5 was solved using x-ray diffraction at a high resolution of 1.6 Å. This ensured the detailed representation of the water-mediated hydrogen-bonding network [45]. 3ZS5 has also being used in a previous computational study [70] and it is bound to the inhibitor SB 203580 [45]. The structure is a monomer consisting of 362 residues and is made up of two domains comprising N- and C-terminal domains. The N-terminal domain is composed of largely β-sheets, while the C-terminal domain is essentially helical [71]. The catalytic ATP binding site is found between the two domains (Figure 1). This site is characterized by a peptide flip, resulting in the presentation of two hydrogen-bond donors in the ATP pocket, a clear distinction from the acceptor-donor-acceptor pattern normally present in the ATP pocket of other protein kinases [45]. One large binding cavity with a surface area of 1040.309 Å^2^ and volume 1365.552 Å^3^ was predicted via CASTp 3.0 [72].

### 3.2. Docking Protocol Validation

**Figure 2:**
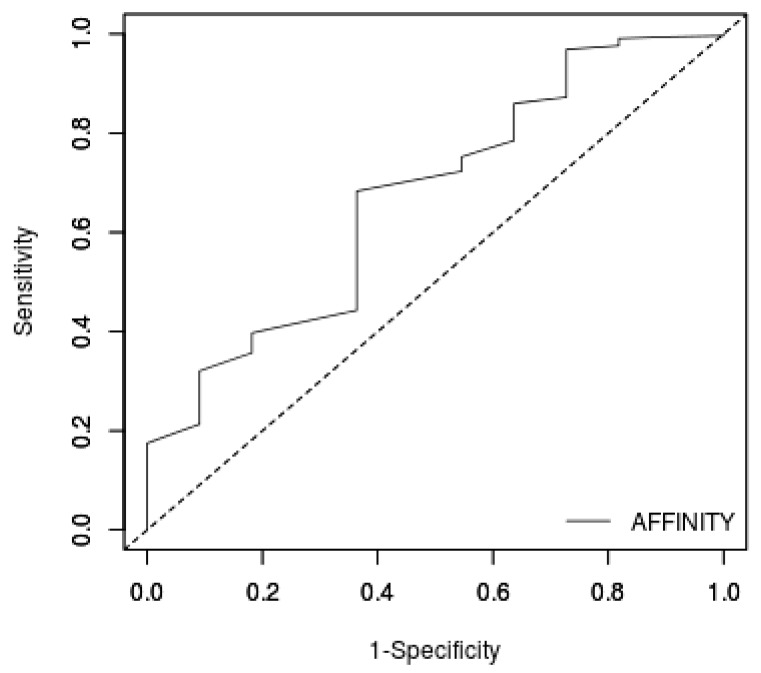
Evaluating the performance of the virtual screening via ROC curve. Binding energies from the screening of inhibitors and decoys against the p38 MAPK receptor were used to generate the curve. An AUC of 0.704 was obtained from the ROC curve.

The docking protocol of AutoDock Vina [60] was validated using the ROC curve. Eleven inhibitors with their respective decoys were screened against the p38 MAPK structure to generate a ROC curve. The ROC curve evaluates the performance of AutoDock Vina to classify inhibitors from decoys [73]. An area under the curve (AUC) value of 1 is considered perfect, while an AUC below 0.5 signifies poor classification [74]. Additionally, an AUC value of 0.7 to 0.8 is considered acceptable, 0.8 to 0.9 is very good, and above 0.9 is excellent [75,76]. The obtained AUC computed from the ROC curve was 0.704 (Figure 2), which is considered acceptable [75]. This indicates that AutoDock Vina reasonably distinguishes between active and inactive compounds of p38 MAPK. AutoDock Vina is more accurate than AutoDock 4 in selecting anti-SARS-CoV-2 M^pro^ compounds [77]. Moreover, AutoDock Vina was employed successfully to screen compounds against p38 MAPK, which were found to inhibit p38 MAPK in vitro [70].

### 3.3. Pre-Filtering of Library and Molecular Docking Studies

A total of 7282 out of the 38,271 natural products were obtained after pre-filtering. These compounds had molecular weights between 250 g/mol and 350 g/mol, and were predicted to be non-toxic. The compounds were screened against the ATP-binding pocket of p38 MAPK and were ranked based on their binding energies. AutoDock Vina combines both empirical scoring functions and knowledge-based potentials to compute the binding energies of compounds [60]. ZINC38321631, a compound annotated in the TCM database, had the lowest binding energy of −12.4 kcal/mol, whereas ZINC5734567 had the highest binding energy of −2.5 kcal/mol. Among the known inhibitors, SB 202190 had the least binding energy of −11.0 kcal/mol, followed by SB 203580 with −10.9 kcal/mol. Losmapimod that is currently undergoing clinical trial had binding energy of −9.3 kcal/mol, whilst that of Dilmapimod was −7.9 kcal/mol. In this study, a threshold of −11.0 kcal/mol was used for selecting hits. Selected compounds had lower binding energies relative to the known inhibitors. A previous study used a threshold of −10.0 kcal/mol for p38 MAPK [78]. Another docking study on the potential inhibitors of p38 MAPK identified a promising compound with a binding energy of −11.1 kcal/mol [79]. A total of 42 compounds and two inhibitors (SB 202190 and SB 203580) were selected for downstream analysis.

### 3.4. Pharmacological Profiling of Hit Compounds

Pharmacological profiling studies are important aspects of the drug development pipeline. The human intestinal absorption, permeability glycoprotein (P-gp) binding, and cytochrome P450 3A4 (CYP450) inhibition were considered [80]. The human intestinal absorption measures the likelihood of absorption for orally administered drugs into the bloodstream. Two of the hits, namely ZINC85550217 and ZINC85543655 were predicted to have low GI absorption. Additionally, 16 out of the 42 compounds including ZINC38321631 were predicted as P-gp substrates with likely decrease in drug bioavailability [81,82]. CYP3A4 is responsible for the metabolism of about 50% of all drugs [83]. The two standard inhibitors (SB 202190 and SB 203580) were predicted as CYP3A4 inhibitors. A total of 17 hits were predicted to be CYP3A4 inhibitors suggesting they may interfere with the metabolism of other drugs. After eliminating compounds predicted to have poor pharmacological profiles, 18 compounds were selected as promising hits (Supplementary Table S1) with none of them violating Lipinski’s rule of five for evaluating drug-likeness.

### 3.5. Visualization and 2-D Representation of Protein-Ligand Interactions

**Figure 3:**
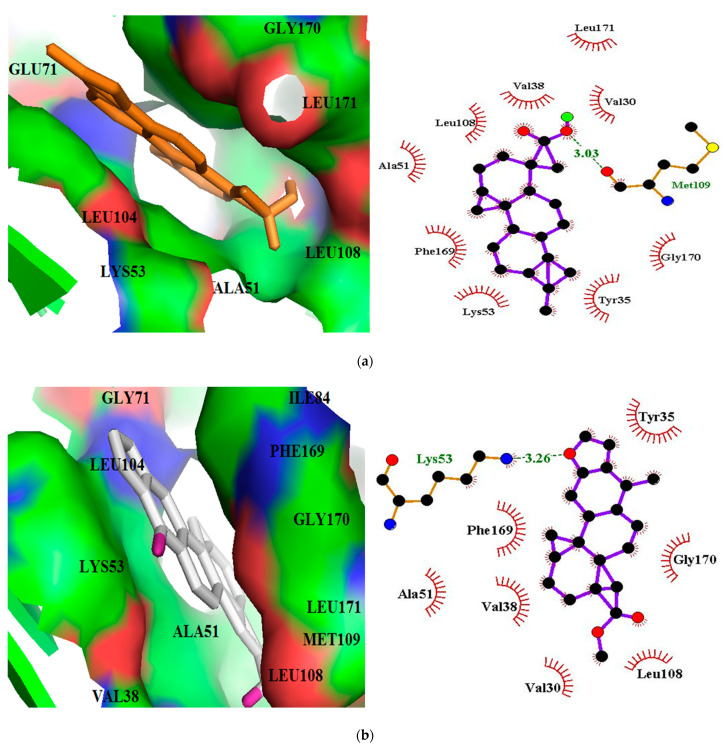

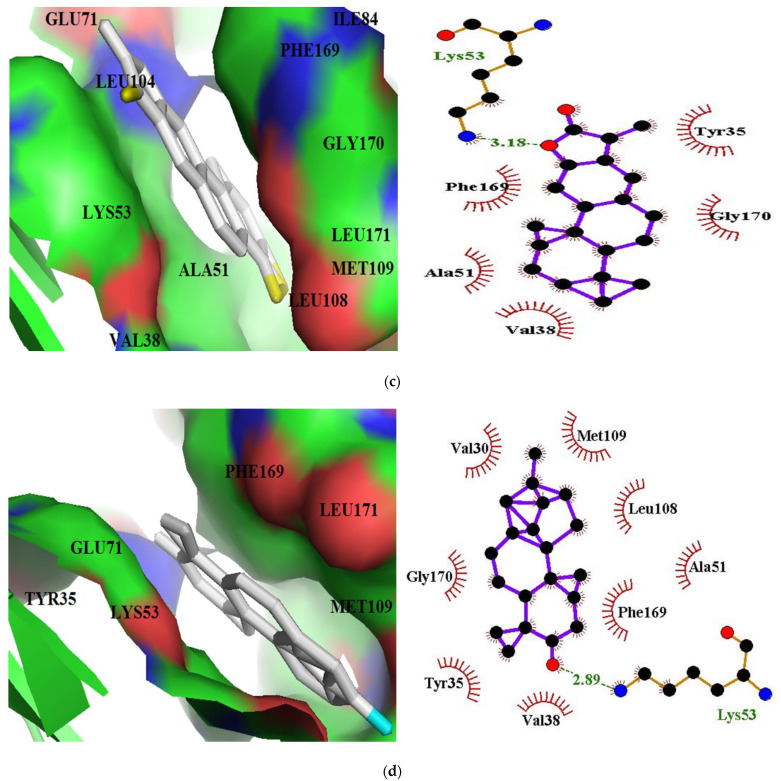
Docking poses and protein-ligand interaction studies of top four hits with the lowest binding energies (**a**) ZINC95486106, (**b**) ZINC95913720, (**c**) ZINC33832090, and (**d**) ZINC95919076 against p38 MAPK structure. The binding pockets are represented as surfaces and the ligands as sticks. In the LigPlot+ representations, the ligands are displayed as purple sticks, hydrophobic contacts are shown as red spoke arcs, and the hydrogen bonds with their respective bond lengths as green.

Hydrogen bonding and hydrophobic contacts are critical in the stabilization of a ligand within the binding pocket of a receptor [84]. All 18 selected hit compounds were observed to bind firmly in the binding pocket of the receptor. ZINC95486106 had the lowest binding energy of −12.1 kcal/mol. It formed hydrogen bonding with Met109 (bond length of 3.03 Å) and hydrophobic contacts with Val30, Ala51, Val38, Leu171, Leu108, Gly170, Lys53, Tyr35 and Phe169 (Figure 3a). Eight other compounds comprising ZINC95913720, ZINC95919076, ZINC33832090, ZINC1691180, ZINC4520996, ZINC4215683, ZINC4023706 and ZINC70454959 formed at least one hydrogen bond with the residues of the binding pocket (Table 1). ZINC4215683 formed three hydrogen bonds with residues Glu71 and Lys53. It also formed hydrophobic contacts with Gly170, Tyr35, Phe169, Val38, Leu86, Leu104, Leu171, Thr106, Leu75 and Val105. Additionally, ZINC95913720 formed a hydrogen bond with Lys53 and hydrophobic contacts with Val30, Ala51, Val38, Leu108, Gly170, Tyr35, Phe169, and Val38 (Figure 3b). ZINC95919076 formed a hydrogen bond with Lys53 and hydrophobic contacts with Val30, Ala51, Val38, Leu108, Gly170, Tyr35, Phe169, Val138, and Met109 (Figure 3d). ZINC1691180 also formed a hydrogen bond with Tyr35 (bond length of 3.17 Å) and hydrophobic contacts with Val30, Ala51, Val38, Phe169, Val38, Met109, Glu71, Leu75, Leu104, Thr106 and Gly31. ZINC4520996 formed hydrogen bonds with Tyr35 and hydrophobic contacts with Ala51, Val38, Val30, Lys53, Gly31, Glu71, Thr106, Phe169, Leu104 and Thr106. ZINC4023706 formed three hydrogen bonds with residues Tyr35 and Gly170, and hydrophobic contacts with Phe169, Leu104, Leu75, Glu71, Thr106, Val38, Ala51, Lys53, Val20 and Gly31. ZINC33832090 had binding energy of −11.8 kcal/mol and formed hydrophobic interactions with Ala51, Val38, Gly170, Tyr35 and Phe169 (Figure 3c). It also interacted with Lys53 via hydrogen bonding of length 3.18 Å (Table 1 and Figure 3c). The known inhibitors SB 202190 and SB 203580 formed only hydrophobic interactions with Tyr35, Val38, Ala51, Lys53, Leu104, Thr106, Leu108, Met109, Phe169, Gly170, and Leu171. Residues worth noting are Ala51, Val38 and Phe169, which interacted separately with 15, 17, and 18 molecules, respectively. These three residues can be considered critical in the stabilization of ligands in the binding pocket. These critical residues also interacted with both SB 202190 and SB 203580. Previous studies identified Lys53, Leu108 and Met109 as crucial residues [45,71,85,86], whilst another identified Phe169 as important [87].

### 3.6. Biological Activity Prediction

The prediction of activity spectra for substances (PASS) [64] was employed to predict relevant biological activities of the hits. PASS utilizes structural-activity relationship to predict the potential biological activities and mechanisms. When probable activity (Pa) is greater than the probable inactivity (Pi) for a particular compound activity and Pa > 0.3, it is essential to test in vitro the predicted activity [57,88]. The biological activities that were considered included anti-inflammation, kinase inhibition, antiviral, platelet aggregation inhibition and immunosuppression. PASS predictions are usually corroborated experimentally [89,90]. ZINC5519433 was predicted as a platelet aggregation inhibitor (Pa = 0.509 and Pi = 0.008), and antiviral (rhinovirus) with Pa = 0.470 and Pi = 0.037. Platelet activation accelerates inflammation and can lead to disseminated intravascular coagulation, a condition observed in critically ill COVID-19 patients [91,92]. ZINC5733756 was predicted as antiviral (influenza) with Pa = 0.755 and Pi = 0.004, anti-inflammatory (Pa = 0.694 and Pi = 0.017), and antiviral (rhinovirus) with Pa = 0.599 and Pi = 0.006. ZINC4023706 was predicted to be antiviral (influenza) with Pa = 0.764 and Pi = 0.004, anti-inflammatory (Pa = 0.693 and Pi = 0.017), and antiviral (rhinovirus) with Pa = 0.554 and Pi = 0.011. ZINC95486106 was predicted as anti-inflammatory (Pa = 0.694 and Pi = 0.017), antiviral (influenza) with Pa = 0.716 and Pi = 0.005, Beta-adrenergic receptor kinase inhibitor (Pa = 0.823, Pi = 0.011), G-protein-coupled receptor (GPCR) kinase inhibitor (Pa = 0.823 and Pi = 0.011), and RNA-directed RNA polymerase inhibitor (Pa = 0.518 and Pi = 0.006). More so, ZINC1691180 and ZINC4520996 were revealed as anti-inflammatory (Pa = 0.645 and Pi = 0.024), antiviral (rhinovirus) with Pa = 0.525 and Pi = 0.017, and antiviral (influenza) with Pa = 0.748 and Pi = 0.004. ZINC1691180 has been shown to elicit anti-inflammatory response in mice [93].

### 3.7. The Rationale for the Selection of Compounds

A lead compound is a molecule that is likely to be of therapeutic relevance and may be modified to improve potency, selectivity, and pharmacological profiles [94]. After screening a library of 7282 pre-filtered compounds against the p38 MAPK structure, 42 compounds with binding energies ≤ −11.0 kcal/mol were selected for pharmacological profiling. A total of 18 compounds were selected from this lot as promising hits due to predicted good pharmacological profiles. The protein-ligand interactions of the promising hits were evaluated to identify residues critical for binding. Further biological activity predictions were performed to identify compounds with relevant biological activities and mechanisms of action. The multistage techniques informed the selection of five compounds, namely ZINC5519433, ZINC5733756, ZINC95486106, ZINC1691180, and ZINC4520996 (Table 2). The selected compounds were predicted as having plausible pharmacological profiles including high gastrointestinal absorption, non-Pgp substrates, and non-CYP3A4 inhibitors. Assessment of their predicted biological activities showed all five to have anti-inflammatory and antiviral propensities, with Pa > Pi and Pa > 0.3.

ZINC5519433 had binding energy of −11.6 kcal/mol (Table 1). It formed hydrophobic interactions with residues including Lys53, Thr106 and Phe169, which have been reported in other studies to be critical in ligand binding (Table 1). It is 71% structurally similar to macelignan via DrugBank [95]; a natural compound that attenuates the activation of p38 MAPK and has exhibited anti-inflammatory activity [96]. Previous experimental studies showed ZINC5519433 as a kinase inhibitor and selective to c-Jun *N*-terminal kinases (JNK) [97]. This corroborates our studies on the potential lead-likeness of ZINC5519433. A further lead optimization may likely increase its selectivity towards p38 MAPK [98].

ZINC1691180 and ZINC4520996 were both predicted to have a binding energy of −11.6 kcal/mol (Table 1). Both formed hydrogen bonding with Tyr35, with varying lengths and hydrophobic contacts with some critical residues (Table 1). ZINC1691180 has been reported to evoke anti-inflammatory properties via regulating the production of inflammatory factor TNFα in mice [93]. TNFα mediates the activation of p38 MAPK [99] and obstruction of this pathway with ZINC1691180 could be useful in severe COVID-19 cases. ZINC5733756 had binding energy of −11.1 kcal/mol due to strong intermolecular interaction with residues including Thr106 and Phe169 (Table 1). Moreover, ZINC5733756 is 73% structurally similar to estradiol, a hormone that exerts anti-inflammatory activities by targeting the activation of Nrf2 [100].

Furthermore, ZINC95486106 formed interactions with critical residues namely Lys53, Leu108, and Phe169 contributing to its binding energy of −12.1 kcal/mol. It was also predicted to be GPCR and Beta-adrenergic receptor kinase inhibitors via PASS with Pa > 0.8.

The inhibitory constants (Ki) of the selected compounds and the known inhibitors were calculated (Supplementary Table S2) using a previously described method [57,101]. All the selected compounds had lower predicted Ki values than all the known inhibitors used herein (Supplementary Table S2), indicating their inhibitory potential [102]. For the selected compounds, ZINC95486106 was predicted to have the lowest Ki value of 1.348 nM while ZINC57337556 had the highest Ki value of 7.291 nM. ZINC1691180, ZINC5519433, and ZINC4520996 had the same predicted Ki value of 3.135 nM (Supplementary Table S2). For the known inhibitors, the predicted Ki values ranged from 8.63 nM to 1.62 µM (Supplementary Table S2). The known inhibitors were experimentally determined to have IC_50_ values ranging from 3.7 to 41.2 nM (Supplementary Table S2) [103,104,105,106,107]. ARRY-797 with the lowest IC_50_ value of 4.5 nM had binding energy of −9.5 kcal/mol, which is higher than that of SB 20358 (−10.9 kcal/mol) with the highest IC_50_ value 41.2 nM.

### 3.8. Molecular Dynamics Simulation of Selected Compounds

A 100 ns MD simulation was performed on eight structures consisting of the unbound protein, the complexes of the five selected compounds, and the two known p38 MAPK inhibitors SB 203580 and SB 202190. This was aimed at probing the structural stability and conformational changes in physiological conditions [108]. The parameters evaluated were the root mean square deviation (RMSD), the radius of gyration (Rg), and the conformational changes with time.

**Figure 4:**
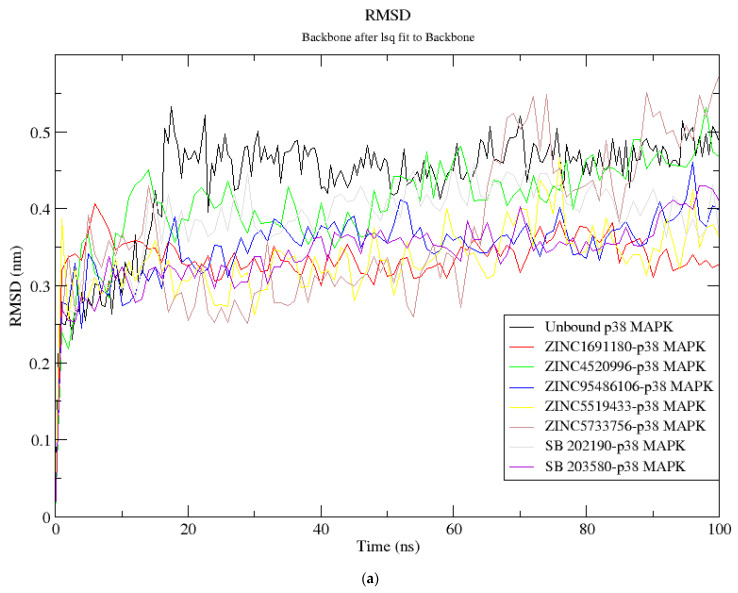

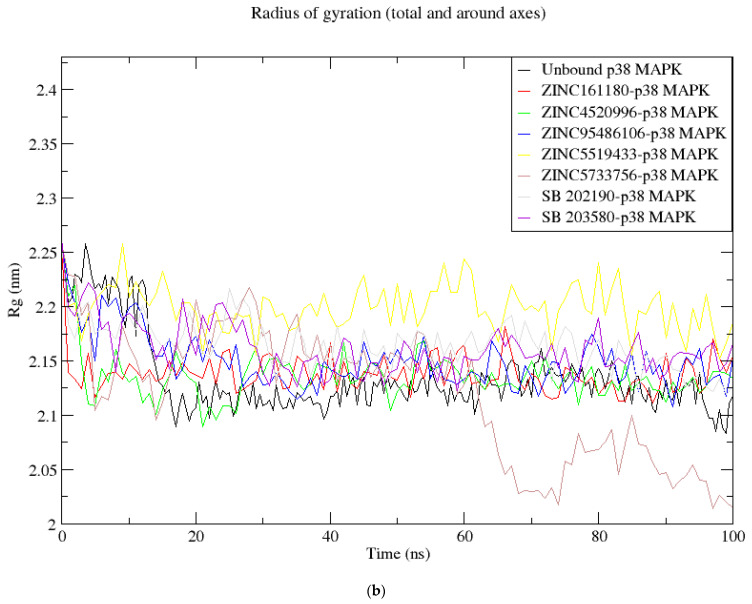
Molecular dynamics simulations graphs (**a**) RMSD versus time (ns) and (**b**) Rg versus time (ns). In (**a**,**b**), the unbound p38 MAPK protein (3ZS5), ZINC1691180, ZINC4520996, ZINC5519433, and ZINC5733756, SB 202190, and SB 203580–p38 MAPK complexes are shown as black, red, green, blue, yellow, brown, ash and purple, respectively.

The RMSD is a metric used to assess the stability of a protein structure [109]. An RMSD plot of the p38 MAPK-ligand complexes that tend to converge is indicative of a stable and well-equilibrated system [110]. Considering the p38 MAPK-ZINC5519433 RMSD plot, a steady rise to 0.38 nm was observed during the 1 ns of simulation. It then descended and maintained an average RMSD of 0.3 nm (Figure 4a). Moreover, the RMSD of the p38 MAPK-ZINC5733756 complex gradually rose to 0.42 nm around 15 ns and quickly dropped to an average RMSD of 0.3 nm around 17 ns (Figure 4a). It maintained the average RMSD of 0.3 nm till about 62 ns, where a sharp rise to an average of 0.45nm was observed till the end of the 100 ns simulation period (Figure 4a). The p38 MAPK-ZINC4520996 complex experienced a rise in RMSD to 0.45 nm around 13 ns and maintained stability till the end of the simulation time (Figure 4a). An initial rise to 0.40 nm during the first 6 ns was observed for the p38 MAPK-ZINC1691180 complex (Figure 4a). The RMSD dropped to about 0.32 nm around 20 ns and was maintained till the 100 ns period (Figure 4a). Additionally, the RMSD plot for p38 MAPK-ZINC95486106 revealed stability after 15 ns, with an average RMSD of 0.33 nm (Figure 4a). Considering the known inhibitors, average RMSDs of 0.35 nm and 0.38 nm were observed for the SB 203580 and SB 202190-p38 MAPK complexes, respectively. The p38 MAPK complexes of ZINC5519433, ZINC95486106, ZINC1691180, ZINC4520996, SB 203580 and SB 202190 had lower average RMSDs relative to the unbound protein (Figure 4a). This may suggest stability induced by ligand binding.

In a pharmacophore-guided study to identify p38 MAPK inhibitors, a 10 ns MD simulation was performed on the most potent molecule (compound 48) and the protein complex [111]. It was observed that the RMSD ranged between 0.5 to 2.5 Å (0.05 to 0.25 nm) [111]. Other MD simulation studies on p38 MAPK-ligand complexes reported RMSD values up to about 0.6 nm [112,113] consistent with the results presented herein. The Rg was measured to evaluate the compactness of the protein systems during the MD simulation [114]. The Rg of p38-MAPK-ZINC5519433 fell from 2.25 nm to 2.20 nm during the first 5 ns and it further rose and peaked at 2.26 nm around 9 ns. It then fluctuated over the remaining time with an average Rg of 2.20 nm (Figure 4b). For the p38 MAPK-ZINC5733756 complex, an initial Rg of 2.2 nm was obtained and it then fluctuated around 2.15 nm until 60 ns, where it dropped to 2.02 nm. The p38 MAPK-ZINC4520996, p38 MAPK-ZINC1691180, p38 MAPK-ZINC95486106, p38 MAPK-SB 203580, and p38 MAPK-SB 202190 complexes were observed to be stable over the simulation time with average Rg of 2.12 nm, 2.13 nm, 2.13 nm, 2.14 nm, and 2.15 nm, respectively. These were close to the average Rg of 2.12 nm exhibited by the unbound protein. A stably folded protein maintains a reasonable steady radius of gyration throughout the simulation [114].

**Figure 5:**
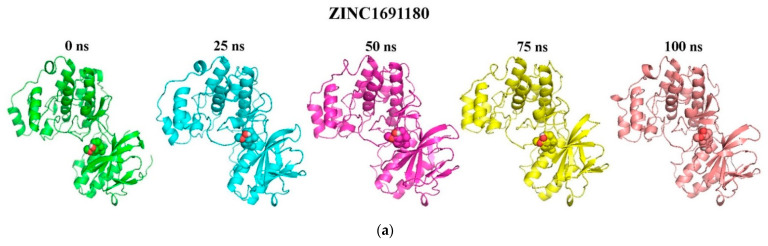

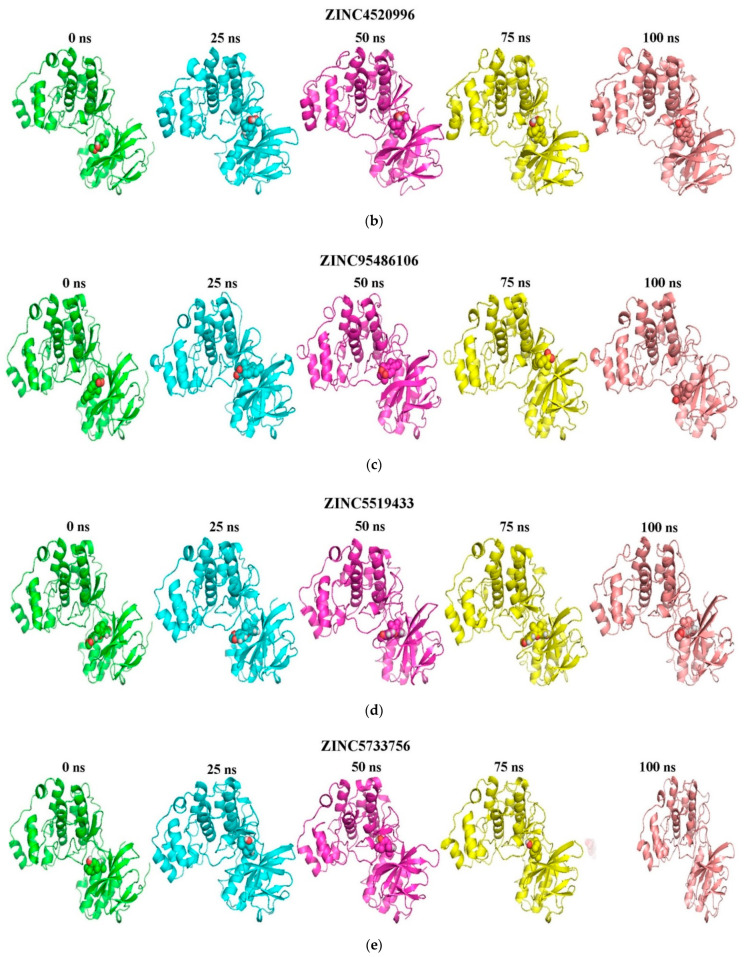
Snapshots at 25 ns intervals ((time step = 0, 25, 50, 75, and 100 ns) for the binding modes of the ligand-p38 MAPK complexes. The cartoon representation shows (**a**) ZINC1691180, (**b**) ZINC4520996, (**c**) ZINC95486106, (**d**) ZINC5519433 and (**e**) ZINC5733756- p38 MAPK complexes. The ligands are represented as spheres and the protein as cartoons. All the ligands were observed to bind stably in the ATP binding pocket.

**Figure 6:**
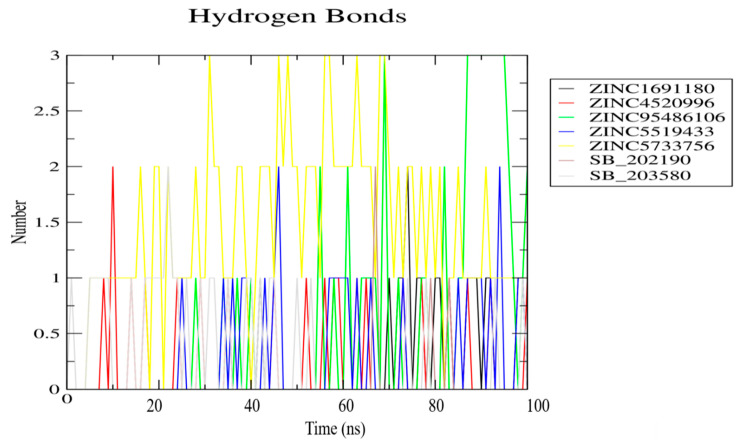
The total number of hydrogen bonds formed between the selected compounds and the protein structure. ZINC1691180, ZINC4520996, ZINC95486106, ZINC5519433, ZINC5733756, SB 202190, and SB 203580-p38 MAPK complexes are represented as black, red, green, blue, yellow, brown and ash, respectively.

The distance between the centre of mass of a protein and an inhibitor influences the molecular interactions, which in turn play a crucial role in the structural stability of the protein-ligand complexes [115]. Snapshots at 25 ns intervals (time step = 0, 25, 50, 75, and 100 ns) were generated to elucidate the position and binding modes of the ligands during the 100 ns MD simulation period (Figure 5 and Supplementary Figure S1). In the snapshot analysis, all the ligands were observed to stably bind in the ATP binding pocket of the p38 MAPK protein throughout the 100 ns simulation time. Moreover, the number of hydrogen bonds over the simulation time with a distance cut-off of 0.35 nm was also generated (Figure 6). The 2D protein-ligand interaction profiles of the snapshot frames of the protein-ligand complexes at time intervals of 25 ns were also generated to study the interactions during the 100 ns simulation period (Supplementary Figures S2 and S3). For the p38 MAPK-ZINC1691180 complex, the hydrogen bond of length 3.17 Å with Tyr35 before MD (Supplementary Figure S2a) was lost at 25 ns and was not formed again throughout the simulation time. However, at 75 ns, ZINC1691180 formed a new hydrogen bond with Ser32 of bond length 2.96 Å, which was lost at 100 ns (Figure S3a). For the p38 MAPK-ZINC4520996 complex, the hydrogen bonding with Tyr35 pre-MD was not present at 25 ns (Supplementary Figures S2b and S3b). However, at 50 ns, ZINC4520996 formed a new hydrogen bond with Asp112 of bond length 3.01 Å (Supplementary Figure S3b). The hydrogen bond with Asp112 was lost at 75 ns and re-formed at 100 ns with a bond length of 3.2 Å (Figure S3b). For the p38 MAPK-ZINC5519433 complex, the ligand was observed to form a hydrogen bond at 25 ns with Gly170 of bond length of 3.15 Å (Supplementary Figure S3d). However, at 50, 75, and 100 ns, there were no hydrogen bonds observed. For the protein-ZINC95486106 complex, the hydrogen bond with Met109 (bond length of 3.03 Å) was lost throughout the simulation time (Supplementary Figures S2c and S3c). However, at 100 ns, two hydrogen bonds were observed with Ser32 of a bond length of 2.73 Å and Lys15 with a bond length of 3.06 Å (Supplementary Figure S3c). For the p38 MAPK-ZINC5733756 complex, ZINC5733756 did not form any hydrogen bond with the protein before MD (Supplementary Figure S2e). At 25 ns, ZINC5733756 was observed to form one hydrogen bond with Asp112 of length 2.86 Å. At 50 ns, it formed two hydrogen bonds with the p38 MAPK protein composed of one with Asp112 (bond length of 2.71 Å) and the other with Asn115 (bond length of 2.70 Å). Hydrogen bonds with Asp112 and Asn115 of lengths 2.65 and 3.14 Å were observed at 75 ns, respectively (Supplementary Figure S3e). At 100 ns, ZINC5733756 maintained only one hydrogen bond of length 2.70 Å with Asp112 (Supplementary Figure S3e). The multiple hydrogen bonds formed indicate the strong interactions between ZINC5733756 and the p38 MAPK protein throughout the simulation period and could influence the activity of the ligand [116].

### 3.9. Evaluation of Selected Compounds via MM-PBSA Calculations

MM-PBSA technique was used to estimate the free binding energies of the seven complexes. This technique addresses limitations with current scoring functions [117,118]. The binding free energy is a more reliable method of evaluating docking studies [77,117,119]. The other parameters computed included the van der Waal, electrostatic, polar, and non-polar solvation energies (Table 3) [120,121]. The selected molecules comprising ZINC5519433, ZINC95486106, ZINC5733756, ZINC1691180 and ZINC4520996 had predicted average free binding energies of −185.122, 30.620, −61.726, −146.008, and −151.561 kJ/mol, respectively. SB 202190 and SB 203580 were predicted to have plausible free binding energies of −166.369 kJ/mol and −236.175 kJ/mol, respectively (Table 3). Since ZINC95486106 was the only selected compound with positive binding free energy, it was eliminated as a potential lead. Optimization of the ligand structure in future studies can improve its affinity to the p38 MAPK protein. The poor binding free energy could largely be due to the high electrostatic energy relative to the other compounds (Table 4) and hence may limit its lead likeness [57]. In all seven compounds assessed, the van der Waal forces contributed favorably to the free binding energies [29,120,121]. However, the polar solvation energy contributed large positive energies to binding in all the complexes [122].

**Figure 7:**
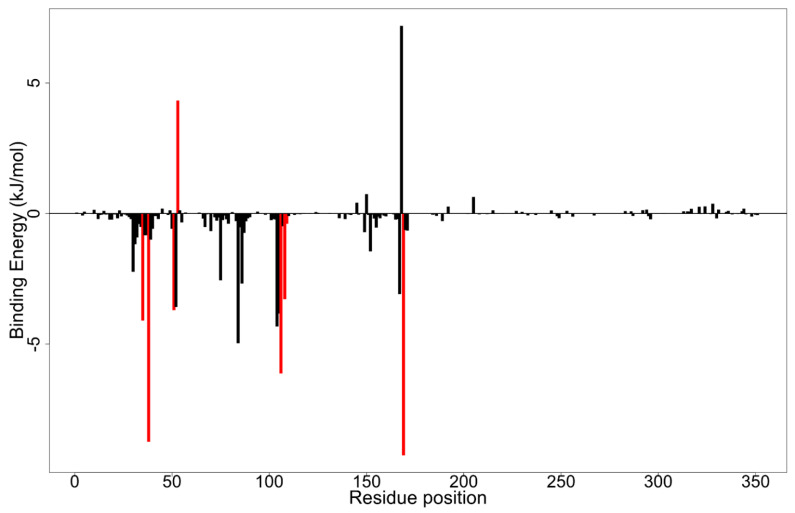
Molecular mechanics Poisson–Boltzmann surface area (MM-PBSA) plot of binding free energy contribution per-residue of ZINC5519433-p38 MAPK complex. Fluctuations of the residues Tyr35, Val38, Ala51, Lys53, Thr106, Leu108, Met109, and Phe169 are colored red.

Additionally, a per-residue decomposition of the binding energy was performed to gain useful insight into important interactions of key residues (Figure 7 and Supplementary Figure S4). Residues contributing binding free energies greater than 5 kJ/mol or less than −5 kJ/mol are worthy of consideration as critical for binding [57]. For the p38 MAPK-ZINC5519433 complex, critical residues Phe169 and Thr106 contributed favorable energies of −9.2470 kJ/mol and −6.1224 kJ/mol, to ligand binding, respectively (Table 4 and Figure 7). Asp168 was found to contribute unfavorable energy of 7.1894 kJ/mol (Figure 7). Moreover, only Phe169 contributed binding free energies of ±5.0 kJ/mol to ZINC5733756 binding (Table 4 and Supplementary Figure S4d). From the energy decomposition plot, Tyr30, Tyr38, Leu167, and Phe169 were predicted as critical residues that contributed favorably to ZINC1691180 binding (Table 4 and Supplementary Figure S4a). However, Lys53 and Asp112 contributed adversely towards ZINC1691180 binding with energies of 8.4199 and 9.7176 kJ/mol, respectively. For ZINC4520996 complex, Lys53, Leu167, Asp168, and Phe169 contributed energies of ±5.0 kJ/mol to binding (Table 4 and Supplementary Figure S4b). Altogether, Lys53 was found to contribute high repulsive positive energies in the p38 MAPK-ZINC5519433, ZINC1691180, ZINC4520996, SB 203580 and SB 202190 complexes (Table 4), unfavorable for ligand binding in the ATP binding pocket. Phe169 contributed favorable energies <−5.0 kJ/mol in all complexes examined except ZINC95486106, corroborating its critical role in ligand binding as suggested in previous studies [87,110].

## 4. Summary and Potential Implication of the Study on COVID-19-Induced Cytokine Storm

The binding of SARS-CoV-2 to the human ACE2 and cell entry has been shown to downregulate ACE2 [18,19], which in turn leads to uncontrolled inflammation [14]. ACE2 is known to convert angiotensin-II to angiotensin (1–7), which binds to the MAS receptor (MASR) to promote vasodilation, vascular protection, anti-fibrosis, anti-proliferation, anti-inflammation, and anti-angiogenesis [123]. Angiotensin-II signals pro-inflammatory, pro-vasoconstrictive, and pro-thrombotic activity through p38 MAPK activation, which is counter-balanced by Angiotensin (1–7) downregulation of p38 MAPK activity [14]. The downregulation of ACE2 leads to poor conversion of angiotensin -II to angiotensin (1–7). Studies on the complications of SARS-CoV-2 infection have reported higher levels of plasma cytokines, a condition known as the cytokine storm [12,13,124]. SARS-CoV was reported to upregulate p38 MAPK activity via a viral protein [20] and SARS-CoV-2 has also been suggested to employ a similar mechanism [14]. The p38 MAPK has thus been identified as critical in the COVID-19 cytokine storms [14,15].

Therefore, this study sought to identify potential p38 MAPK inhibitors which could be explored as useful in ameliorating the cytokine storms in severely ill COVID-19 patients. Pharmacoinformatics-based approaches including molecular docking and dynamics studies predicted four potential lead compounds with good binding affinity to the p38 MAPK protein and negligible toxicity. The predicted biological activities of the selected compounds showed that they possessed anti-inflammatory, kinase inhibition, antiviral, platelet aggregation inhibition, and immunosuppression activities. Dexamethasone, which is an anti-inflammatory compound is used as a treatment option in COVID-19 patients [26]. Moreover, Losmapimod, which is a p38 MAPK inhibitor, is currently undergoing clinical trials for use as a treatment option in severely ill COVID-19 patients (https://clinicaltrials.gov/ct2/show/NCT04511819 (accessed on 8 April 2021)). Herein, ZINC5519433 was predicted as a platelet aggregation inhibitor and anti-rhinovirus. ZINC5733756 was also predicted as an anti-inflammatory, anti-influenza, and anti-rhinovirus. Moreover, ZINC1691180 and ZINC4520996 were predicted to be anti-inflammatory and antiviral. Interestingly, ZINC1691180 has previously been shown to elicit an anti-inflammatory response in mice [93]. The effects of the predicted compounds as potential inhibitors of p38 MAPK to possibly reduce the cytokine storm can be investigated experimentally. The plethora of predicted activities and mechanisms serve as clues that warrant further evaluation of these compounds. The molecules can be optimized as well as used for fragment-based *de novo* drug design of potential novel biotherapeutics. These predicted compounds can help fuel the pace of searching for effective antivirals with anti-inflammatory activity.

## 5. Conclusions

The study used multistage computational techniques to predict four natural product-derived molecules comprising ZINC5733756, ZINC1691180, ZINC4520996 and ZINC5519433, which have the potential to inhibit the p38 MAPK. The compounds were selected based on high binding affinity and plausible binding mechanisms with the p38 MAPK protein structure obtained through molecular dynamics simulations including MM-PBSA. Key residues Tyr38, Ala51 and Phe169 were corroborated as critical for binding, which could guide the design and selection of future p38 MAPK inhibitors. Furthermore, these drug-like compounds were predicted as anti-inflammatory, kinase inhibitors, antiviral, platelet aggregation inhibitors, and immunosuppressive. Since the activation of the p38 MAPK leads to hyperinflammation in severely ill COVID-19 patients, the potential of these molecules to attenuate the cytokine storm can be explored. Further in vitro and in vivo evaluations of the suggested molecules could be undertaken to corroborate the predicted inhibitory activity. The study can serve as a clue for the design of novel p8α MAPK selective inhibitors which may have therapeutic implications in COVID-19-induced cytokine storm.

## Figures and Tables

**Table 1:** Summary of the protein-ligand interactions generated with LigPlot+ for the top 18 hits and 2 inhibitors. The binding energies, number of hydrogen bonds, hydrogen bond interacting residues with their respective bond lengths, and hydrophobic contacts of the compounds are shown.

ZINC ID/Drug Name	Binding Energy (kcal/mol).	Number of Hydrogen Bonds	Hydrogen Bond Residues	Hydrogen Bond Length (Å)	Hydrophobic Contacts
ZINC95486106	−12.1	1	Met109	3.03	Val30, Ala51, Val38, Leu171, Leu108, Gly170, Lys53, Tyr35, Phe169
ZINC95913720	−11.8	1	Lys53	3.26	Val30, Ala51, Val38, Leu108, Gly170, Tyr35, Phe169
ZINC33832090	−11.8	1	Lys53	3.18	Ala51, Val38, Gly170, Tyr35, Phe169
ZINC95919076	−11.7	1	Lys53	2.89	Val30, Ala51, Val38, Leu108, Gly170, Tyr35, Phe169, Val138, Met109
ZINC1691180	−11.6	1	Tyr35	3.17	Val30, Ala51, Val38, Phe169, Glu71, Leu75, Leu104, Thr106, Gly31
ZINC5519433	−11.6				Ala51, Val38, Gly170, Lys53, Tyr35, Phe169, Leu75, Leu86, Ile84, Leu104, Val105, Thr106
ZINC4520996	−11.6	1	Tyr35	3.16	Ala51, Val38, Val30, Lys53, Gly31, Glu71, Thr106 Phe169, Leu104, Thr106
ZINC1531907	−11.6				Ala51, Val38, Gly170, Lys53, Tyr35, Phe169, Leu75, Leu86, Ile84, Leu104, Val105, Thr106
ZINC4098804	−11.6				Ala51, Val38, Gly170, Lys53, Tyr35, Phe169, Leu75, Leu86, Ile84, Leu104, Val105, Thr106
ZINC95919075	−11.5				Ala51, Val30, Gly170, Lys53, Tyr35, Phe169, Val38, Met109, Leu108
ZINC13302897	−11.4				Ala51, Gly170, Lys53, Tyr35, Phe169, Val38,
ZINC4215683	−11.2	3	Glu71, Lys53 [2]	2.81, 3.12, 2.90	Gly170, Tyr35, Phe169, Val38, Leu86, Leu104, Leu171, Thr106, Leu75, Val105
ZINC13302884	−11.2				Val30, Val38, Ala51, Thr106, Leu104, Glu71, Leu75, Lys53, Phe169, Tyr35
ZINC13302890	−11.2				Leu171, Val38, Gly170, Tyr35, Lys53, Phe169, Ala51
ZINC4023706	−11.1	3	Tyr35, Gly170 [2]	3.22, 3.18, 3.17	Phe169, Leu104, Leu75, Glu71, Thr106, Val38, Ala51, Lys53, Val20, Gly31
ZINC5733756	−11.1				Leu75, Ile84, Leu104, Lys53, Phe169, Leu171, Tyr35, Val38, Leu171, Thr106
ZINC70454959	−11.1	1	Lys53	2.95	Tyr35, Gly170, Leu171, la51, Val138, Phe169
ZINC85993836	−11.1				Tyr35, Val38, Ala51, Lys53, Leu75, Ile84, Val105, Leu104, Thr106, Phe169, Gly170
SB 202190	−11.0				Tyr35, Val38, Ala51, Lys53, Leu104, Thr106, Leu108, Met109, Phe169, Gly170, Leu171
SB 203580	−10.9				Tyr35, Val38, Ala51, Lys53, Leu104, Thr106, Leu108, Met109, Val30, Phe169, Gly170, Leu171

**Table 2:** A list of selected compounds with their 2D structures and common /IUPAC names.

Ligand ID	Common/IUPAC Name	2D Structure
ZINC5519433	Zuihonin A	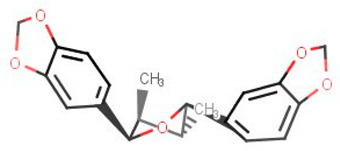
ZINC5733756	8,11,13-Abietatriene-3beta-ol	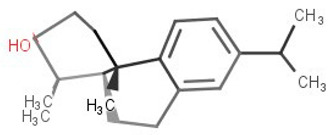
ZINC95486106	(1S,2aS,2bR,4aS,5R,8aS,8bR,10aR)-1,5,8a-trimethyl-hexadecahydrocyclobuta[a]phenanthrene-5-carboxylic acid	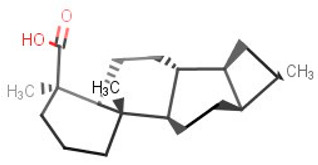
ZINC1691180	Methyl dehydroabietate	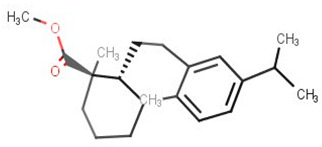
ZINC4520996	Methyl (1S,4aR,10aS)-1,4a-dimethyl-7-propan-2-yl-2,3,4,9,10,10a-hexahydrophenanthrene-1-carboxylate	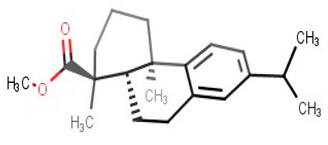

**Table 3:** The energy contributions for the protein-ligand complexes based on the MM-PBSA computations.

Name	Electrostatic Energy (kJ/mol)	Van Der Waal Energy (kJ/mol)	Polar solvation Energy (kJ/mol)	Non-Polar Solvation Energy (kJ/mol)	Binding Energy (kJ/mol)
ZINC5733756	−15.833 ± 16.423	−118.111 ± 77.765	47.787 ± 28.117	−9.689 ± 8.856	−95.846 ± 74.930
ZINC5519433	−2.575 ± 7.169	−222.685 ± 10.572	58.332 ± 15.055	−18.194 ± 0.535	−185.122 ± 21.347
ZINC95486106	75.738 ± 77.290	−86.044 ± 50.930	49.672 ± 75.935	−8.747 ± 4.846	30.620 ± 42.755
ZINC1691180	−12.086 ± 5.07	−180.593 ± 17.415	63.084 ± 16.371	−16.413 ± 0.870	−146.008 ± 17.297
ZINC4520996	2.277 ± 5.466	−203.698 ± 17.665	66.835 ± 11.789	−16.976 ± 0.738	−151.561 ± 22.622
SB 202190	−2.041 ± 3.990	−209.281 ± 15.503	63.370 ± 16.798	18.417 ± 0.895	−166.369 ± 19.355
SB 203580	−5.094 ± 3.533	−280.578 ± 10.532	70.328 ± 22.075	−20.831 ± 1.546	−236.175 ± 26.555

**Table 4:** A table showing the per-residue energy contributions of the critical residues interacting with the ligands. The energies were calculated from the MM-PBSA computations. The energy values are presented in kJ/mol.

Residue	ZINC5733756	ZINC5519433	ZINC95486106	ZINC1691180	ZINC4520996	SB 202190	SB 203580
Tyr35	−0.0810	−4.0969	−0.0881	−1.9196	−0.5451	−1.6995	−3.0867
Val38	−3.8266	−8.7247	−0.7025	−7.6582	−4.7471	−11.3682	−4.3341
Ala51	−1.7431	−3.5809	−0.6868	−2.7066	−4.0012	−5.1339	−2.7369
Lys53	4.9439	4.3252	−12.3692	8.4199	13.3187	12.708	14.7836
Thr106	−0.6584	−6.1224	0.0030	0.5693	−0.1483	−2.5140	−6.7417
Leu108	−3.1269	−3.2785	−0.7509	−4.0640	−7.2590	−3.8154	−0.0608
Met109	−4.2382	−0.3931	−2.1912	−0.2787	−1.8900	−0.4842	−0.7980
Phe169	−5.8140	−9.2470	−1.3285	−14.5954	−6.8001	−14.9162	−10.2842

## Data Availability

Not applicable.

## Supplementary Materials

The following are available online at https://www.mdpi.com/article/10.3390/biom11050653/s1, Table S1: Predicted pharmacokinetics of the 18 selected hits and two inhibitors. The profiles used were gastrointestinal (GI) absorption, cytochrome P450 (CYP) 3A4 and permeability glycoprotein (P-gp) substrates. Also included is the number of violations of Lipinski’s rule of five, Table S2: Predicted inhibitory constants (Ki) and binding energy of the selected compounds and those of the known inhibitors. Also, included are the experimentally determined inhibition constants of known inhibitors., Figure S1: Snapshots at 25 ns interval (time step = 0, 25, 50, 75 and 100 ns) for the binding modes of the (a) SB 202190, and (b) SB 203580 ligand-p38 MAPK complexes, Figure S2: 2D representation of the protein-ligand complexes before MD for (a) ZINC1691180, (b) ZINC4520996, (c) ZINC95486106, (d) ZINC5519433, and (e) ZINC5733756-p38 MAPK complexes, Figure S3: 2D representation of the protein-ligand complexes at 25 ns, 50 ns, 75 ns and 100 ns for (a) ZINC1691180, (b) ZINC4520996, (c) ZINC95486106, (d) ZINC5519433 and (e) ZINC5733756 (f) SB 202190 and (g) SB 203580- p38 MAPK complexes, Figure S4: Molecular mechanics Poisson-Boltzmann surface area (MM-PBSA) plot of binding free energy contribution per-residue for (a) ZINC1691180, (b) ZINC4520996, (c) ZINC95486106, (d) ZINC5733756, (e) SB 202190, and (f) SB 203580-p38 MAPK complexes. Fluctuations of the residues Tyr35, Val38, Ala51, Lys53, Thr106, Leu108, Met109 and Phe169 are colored red.
