# Supplementary material for: Computational Identification of Potential Anti-Inflammatory Natural Compounds Targeting the p38 Mitogen-Activated Protein Kinase (MAPK): Implications for COVID-19-Induced Cytokine Storm
Source: Biomolecules. 2021 Apr 29;11(5):653. doi: 10.3390/biom11050653 (PMC8146027; doi:10.3390/biom11050653)
Supplement: Supplementary file 1 [file biomolecules-11-00653-s001.zip › biomolecules-1145090-supplementary.pdf]

biomolecules

Supplementary Materials

Computational Identification of Potential Anti-Inflammatory Natural Compounds Targeting the p38 Mitogen-Activated Protein kinase (MAPK): Implications for COVID-19-Induced Cytokine Storm

Seth O. Asiedu [1], Samuel K. Kwofie [2,3,*], Emmanuel Broni [2] and Michael D. Wilson [1,4]

[1] Department of Parasitology, Noguchi Memorial Institute for Medical Research, College of Health Sciences, University of Ghana, Legon, Accra, Ghana; soasiedu@noguchi.ug.edu.gh (S.O.A); MWilson@noguchi.ug.edu.gh (M.D.W)
[2] Department of Biomedical Engineering, School of Engineering Sciences, College of Basic and Applied Sciences, University of Ghana, Legon, Accra, Ghana; ebroni002@st.ug.edu.gh
[3] West African Centre for Cell Biology of Infectious Pathogens, Department of Biochemistry, Cell and Molecular Biology, College of Basic and Applied Sciences, University of Ghana, Accra, Ghana
[4] Department of Medicine, Loyola University Medical Center, Maywood, IL 60153, USA
* Correspondence: skkwofie@ug.edu.gh

Citation: Asiedu, S.O.; Kwofie, S.K.; Broni, E.; Wilson, M.D. Computational Identification of Potential Anti-Inflammatory Natural Compounds Targeting the p38 Mitogen-Activated Protein kinase (MAPK): Implications for COVID-19-Induced Cytokine Storm. *Biomolecules* **2021**, *11*, 653. https://doi.org/10.3390/biom11050653

Academic Editor: José L. Medina-Franco

Received: 27 February 2021
Accepted: 26 April 2021
Published: 29 April 2021

Publisher's Note: MDPI stays neutral with regard to jurisdictional claims in published maps and institutional affiliations.

Copyright: © 2021 by the authors. Submitted for possible open access publication under the terms and conditions of the Creative Commons Attribution (CC BY) license (http://creativecommons.org/licenses/by/4.0/).

Table S1. Predicted pharmacokinetics of the 18 selected hits and two inhibitors. The profiles used were gastrointestinal (GI) absorption, cytochrome P450 (CYP) 3A4 and permeability glycoprotein (P-gp) substrates. Also included is the number of violations of Lipinski's rule of five.

Name	GI absorption	Pgp substrate	CYP3A4 inhibitor	Lipinski #violations
ZINC95486106	High	No	No	1
ZINC95913720	High	No	No	0
ZINC33832090	High	No	No	1
ZINC95919076	High	No	No	1
ZINC1691180	High	No	No	1
ZINC5519433	High	No	No	0
ZINC4520996	High	No	No	1
ZINC1531907	High	No	No	0
ZINC4098804	High	No	No	0
ZINC95919075	High	No	No	1
ZINC13302897	High	No	No	1
ZINC4215683	High	No	No	0
ZINC13302884	High	No	No	1
ZINC13302890	High	No	No	1
ZINC4023706	High	No	No	1
ZINC5733756	High	No	No	1
ZINC70454959	High	No	No	0
ZINC85993836	High	No	No	1
SB 202190	High	Yes	Yes	0
SB 203580	High	Yes	Yes	0

Table S2. Predicted inhibitory constants (Ki) and binding energy the selected compounds and those of the known inhibitors. Also, included are the experimentally determined inhibition constants of known inhibitors.

Known Inhibitors used in this study				
Name	Binding energy (kcal/mol)	Inhibitory constant (Ki) based on binding energies	Experimental Inhibitory Constants	PubMedID
SB_202190	-11	8.63 nM	40 nM (IC_{50})	PMID: 9207191
SB 203580	-10.9	10.22 nM	41.2 nM (IC_{50})	PMID: 9207191
Doramapimod	-9.9	55.27 nM	7.1 nM (IC_{50})	PMID: 26279429
Skepinone-L	-9.8	65.44 nM	5 nM (IC_{50})	PMID: 22198732
ARRY-797	-9.5	108.58 nM	4.5 nM (IC_{50})	PMID: 19950901
Losmapimod	-9.3	152.18 nM	8.1 (pKi)	PMID: 19950901
SCIO_469	-9.1	213.29 nM	9 nM (IC_{50})	PMID: 19950901
PH-797804	-8.4	695.22 nM	26 nM (IC_{50})	PMID: 19720877
VX-702	-8.4	695.22 nM	3.7 nM (K_D)	PMID: 19950901
Pamapimod	-8.2	974.41 nM	16 nM (IC_{50})	PMID: 26279429
Dilmapimod	-7.9	1616.83 nM	-	PMID: 19950901

Selected leads		
Name	Binding energy (kcal/mol)	Predicted Ki
ZINC95486106	-12.1	1.348 nM
ZINC1691180	-11.6	3.135 nM
ZINC5519433	-11.6	3.135 nM
ZINC4520996	-11.6	3.135 nM
ZINC5733756	-11.1	7.291 nM

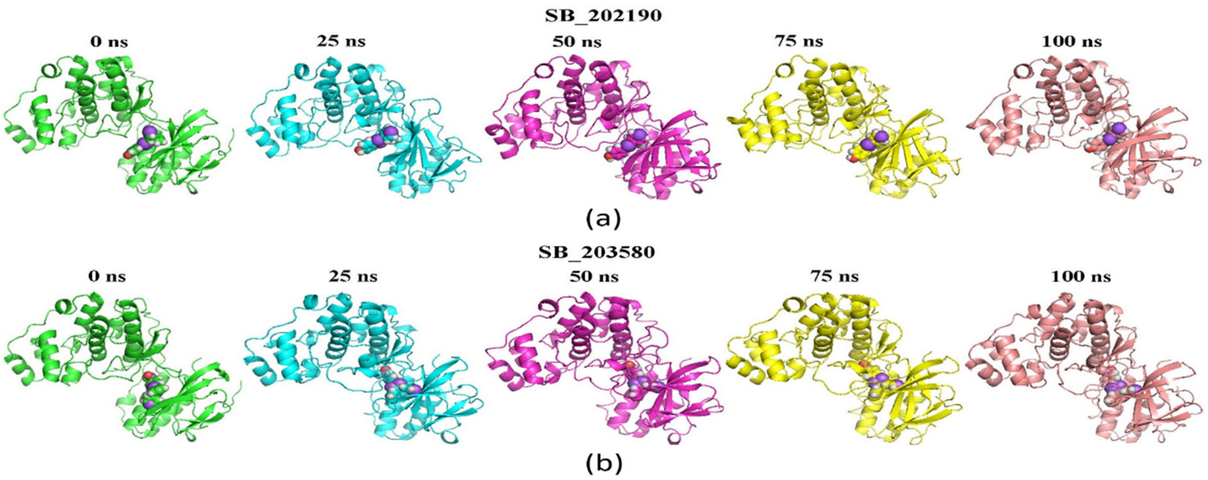

Figure S1. Snapshots at 25 ns interval (time step = 0, 25, 50, 75 and 100 ns) for the binding modes of the (a) SB 202190, and (b) SB 203580 ligand-p38 MAPK complexes.

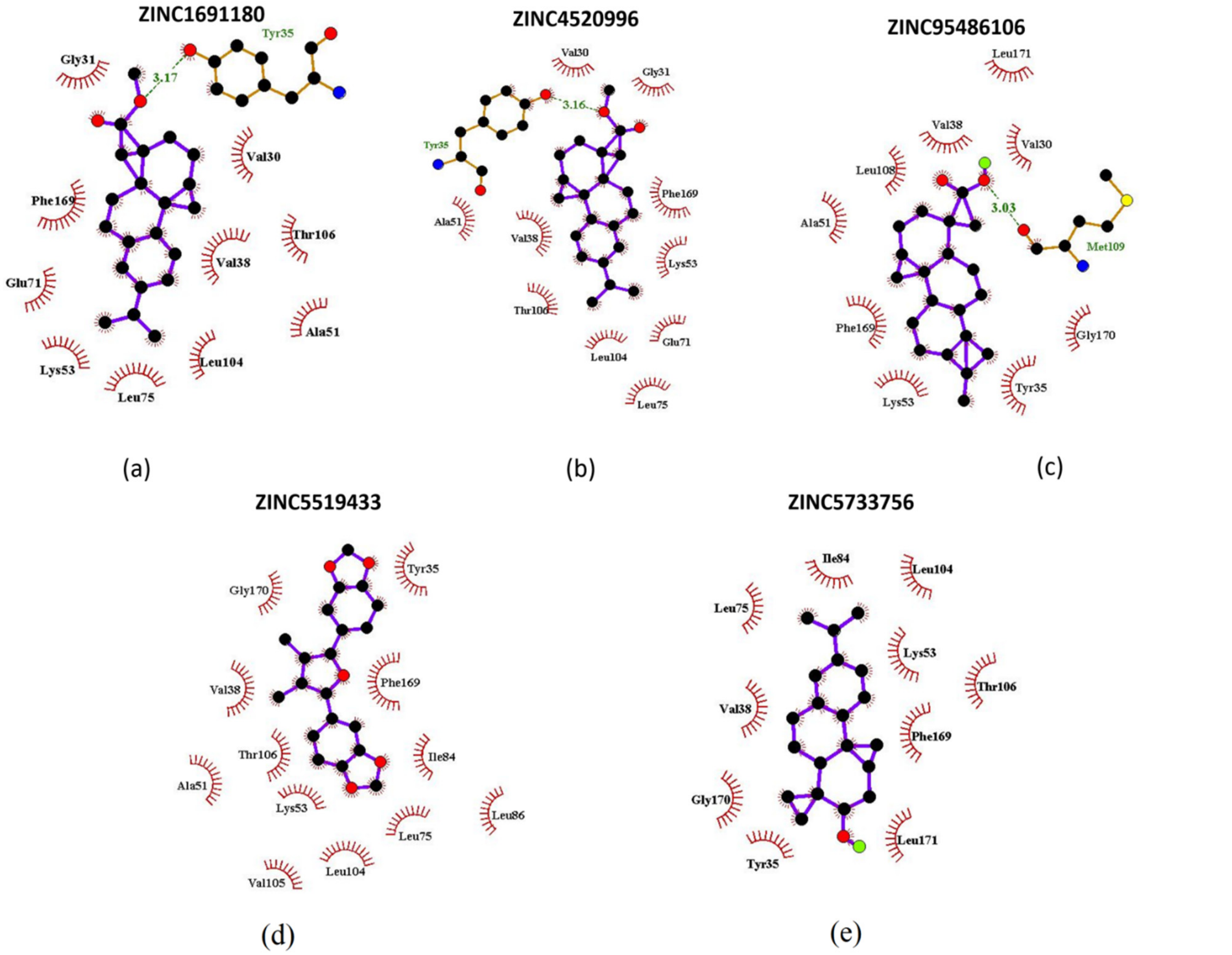

Figure S2. 2D representation of the protein-ligand complexes before MD for (a) ZINC1691180, (b) ZINC4520996, (c) ZINC95486106, (d) ZINC5519433, and (e) ZINC5733756-p38 MAPK complexes.

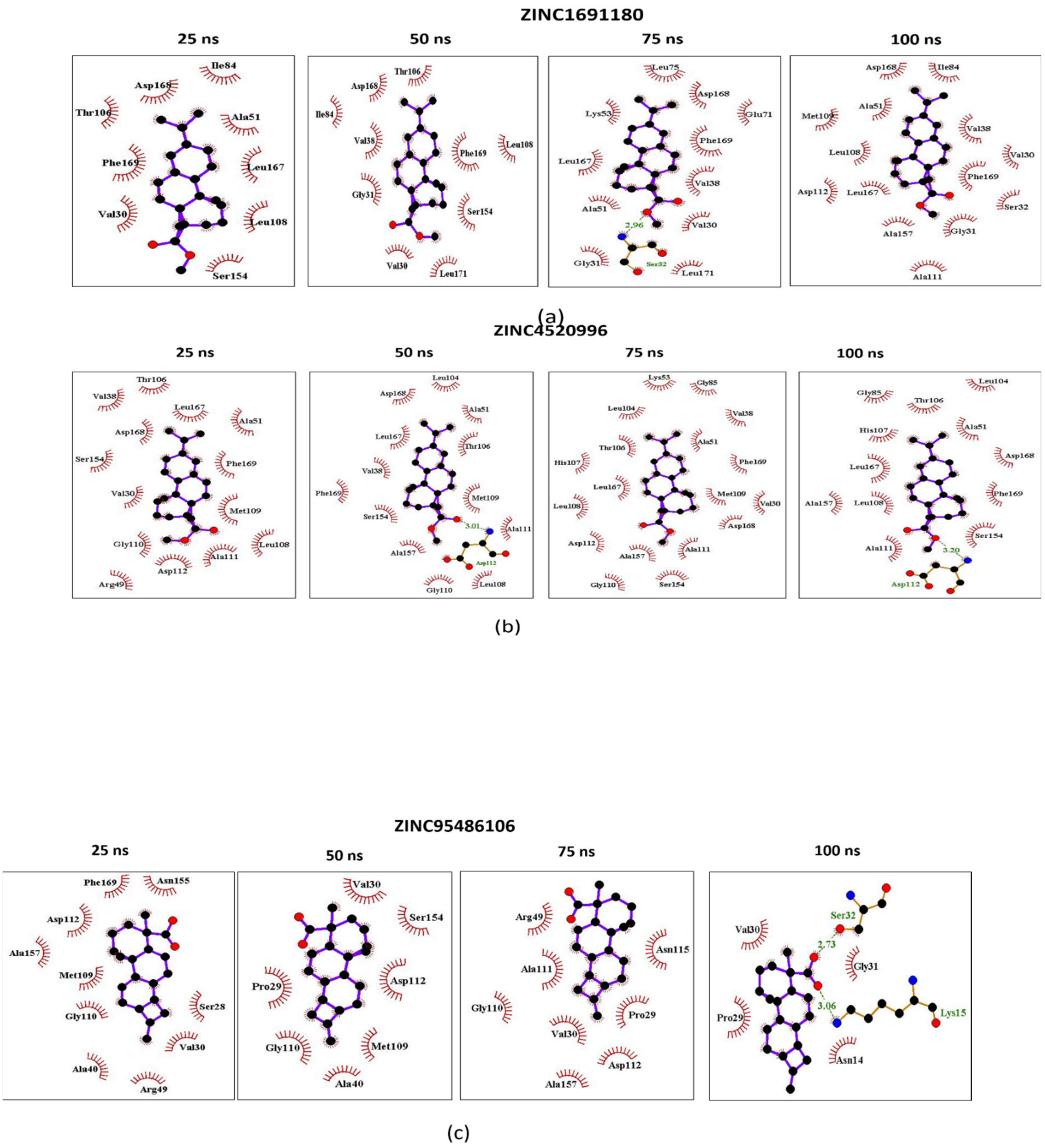
ZINC1691180
25 ns
50 ns
75 ns
100 ns
Ile84
Asp168
Thr106
Ala51
Phe169
Leu167
Val30
Leu108
Ser154
Val38
Gly31
Leu171
Leu75
Lys53
Glu71
2.96
Ser32
Met109
Asp112
Ala157
Ala111
(a)
ZINC4520996
Leu104
Arg49
Gly110
His107
Gly85
3.01
3.20
(b)
ZINC95486106
Asn155
Ser28
Ala40
Pro29
Asn115
2.73
3.06
Lys15
Asn14
(c)

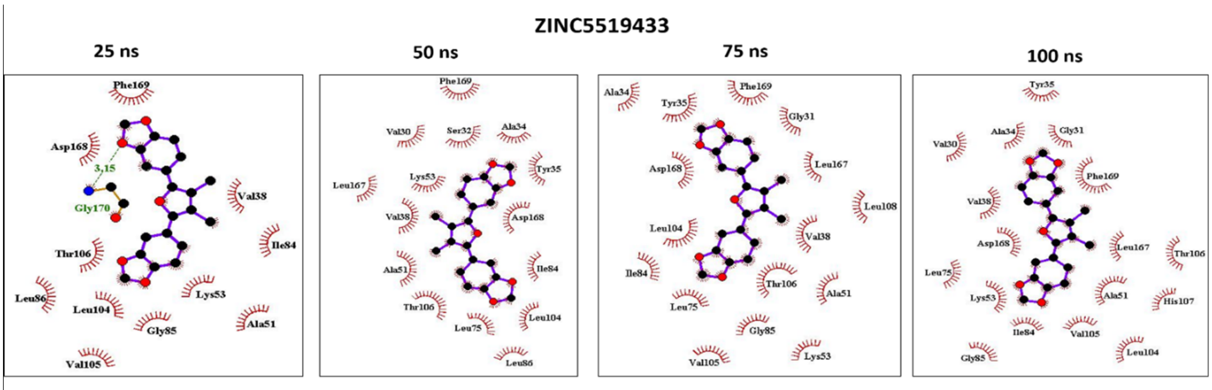
ZINC5519433
25 ns
50 ns
75 ns
100 ns

(d)

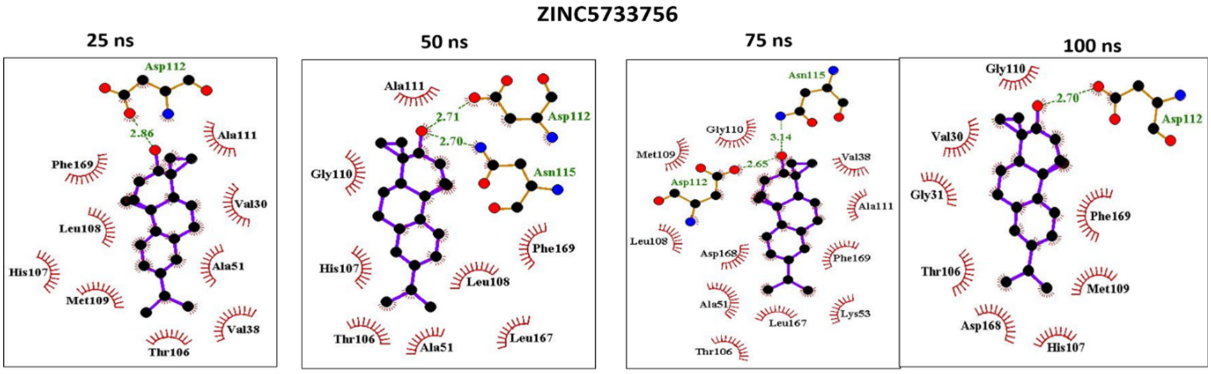
ZINC5733756
25 ns
50 ns
75 ns
100 ns

(e)

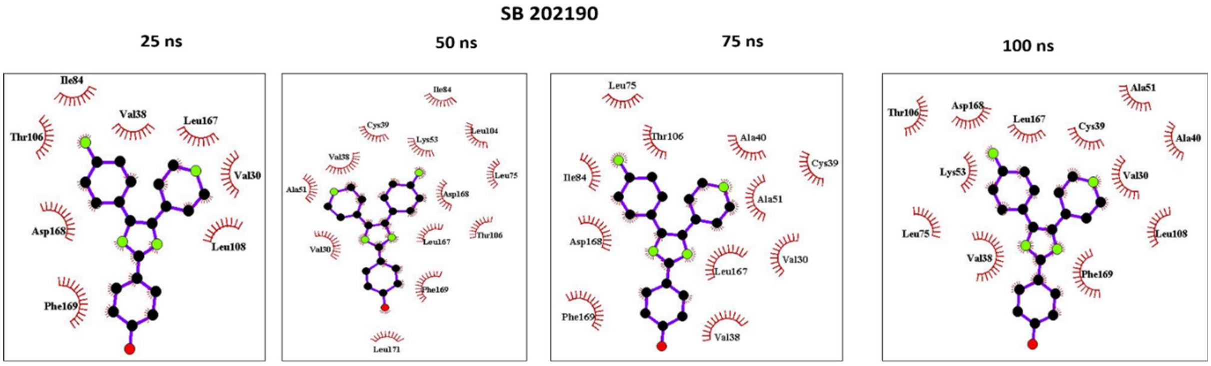
SB 202190
25 ns
50 ns
75 ns
100 ns

(f)

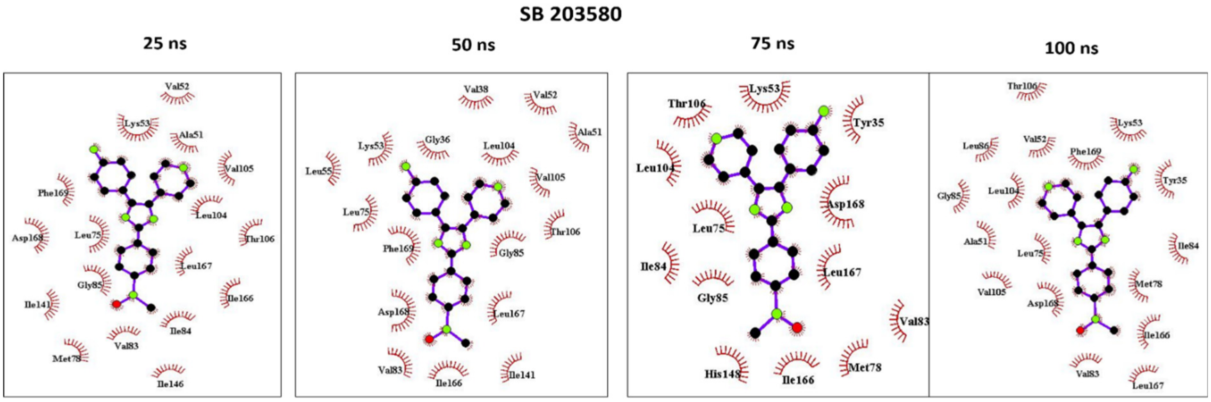

(g)

Figure S3. 2D representation of the protein-ligand complexes at 25 ns, 50 ns, 75 ns and 100 ns for (a) ZINC1691180, (b) ZINC4520996, (c) ZINC95486106, (d) ZINC5519433 and (e) ZINC5733756 (f) SB 202190 and (g) SB 203580- p38 MAPK complexes.

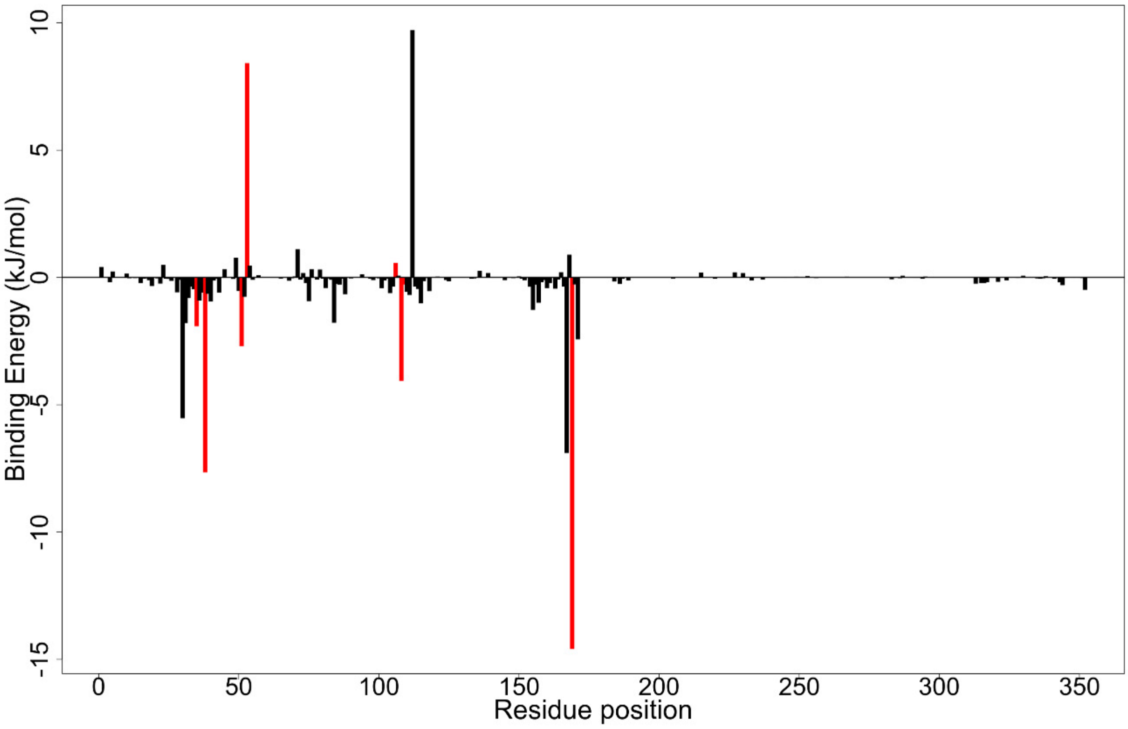

(a)

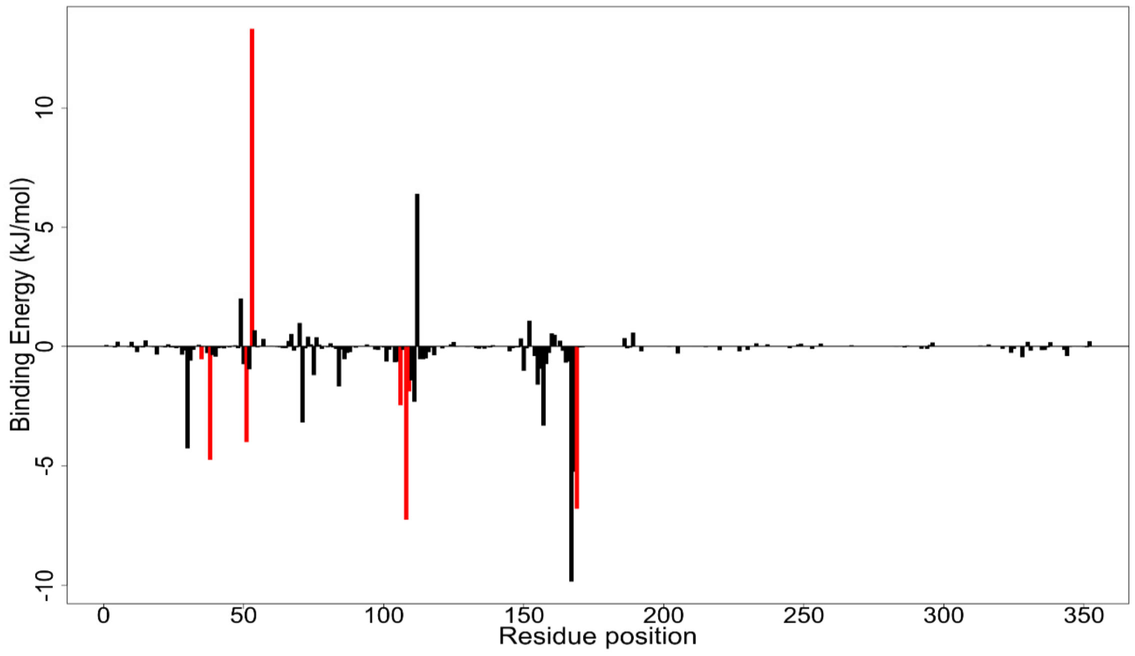
Binding Energy (kJ/mol)
10
5
0
-5
-10
0
50
100
150
200
250
300
350
Residue position

(b)

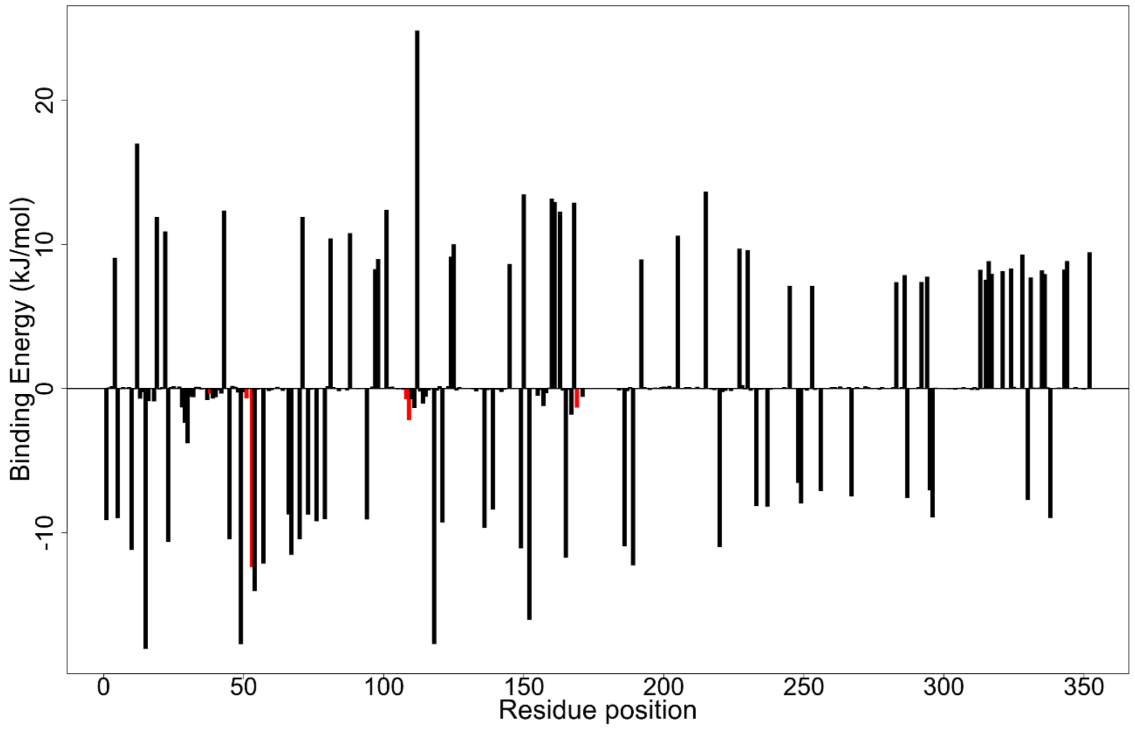
Binding Energy (kJ/mol)
20
10
0
-10
0
50
100
150
200
250
300
350
Residue position

(c)

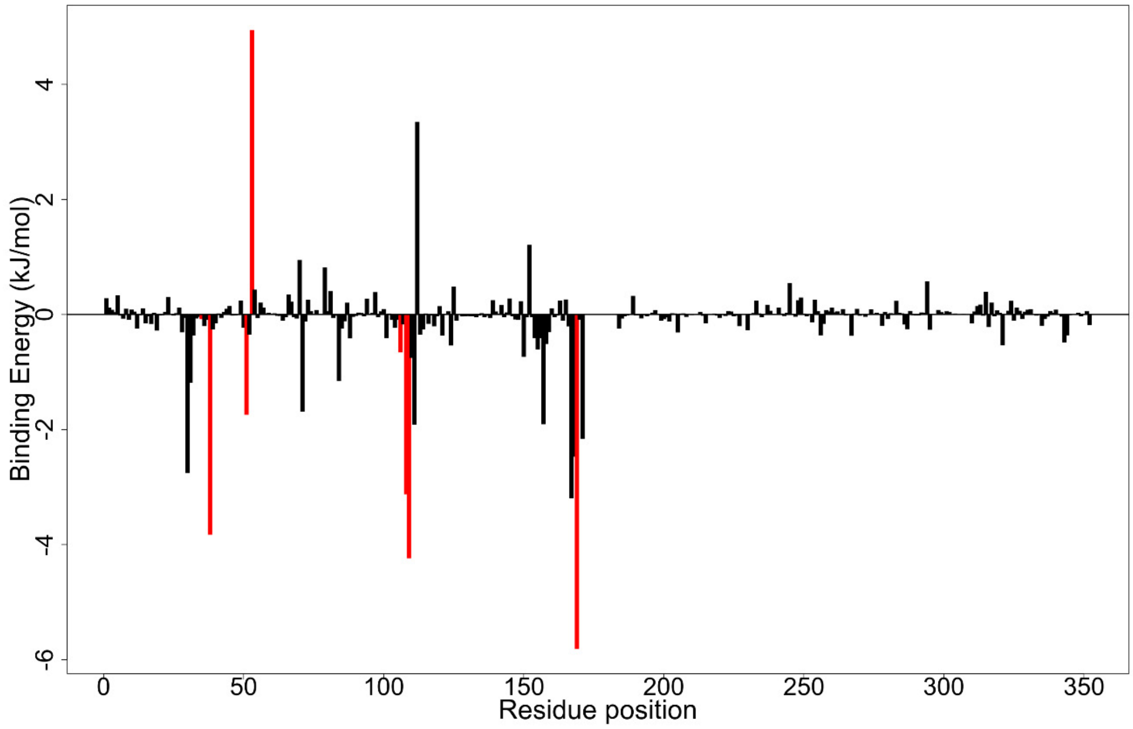
Binding Energy (kJ/mol)
4
2
0
-2
-4
-6
0
50
100
150
200
250
300
350
Residue position

(d)

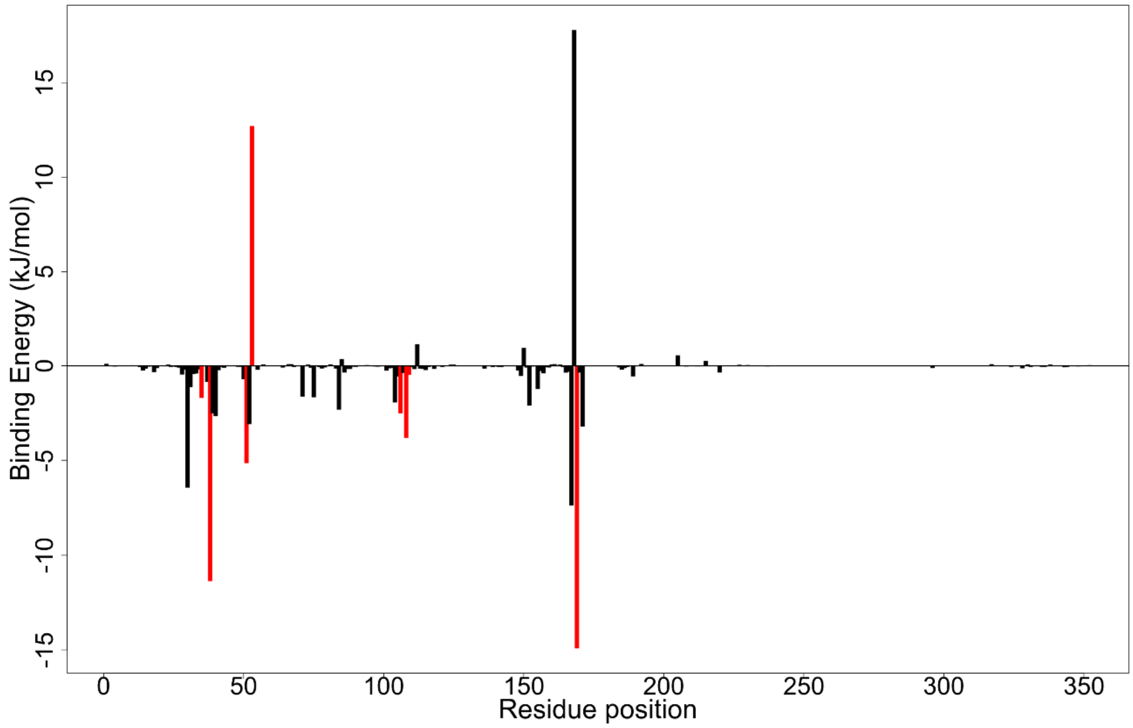
Binding Energy (kJ/mol)
15
10
5
0
-5
-10
-15
0
50
100
150
200
250
300
350
Residue position

(e)

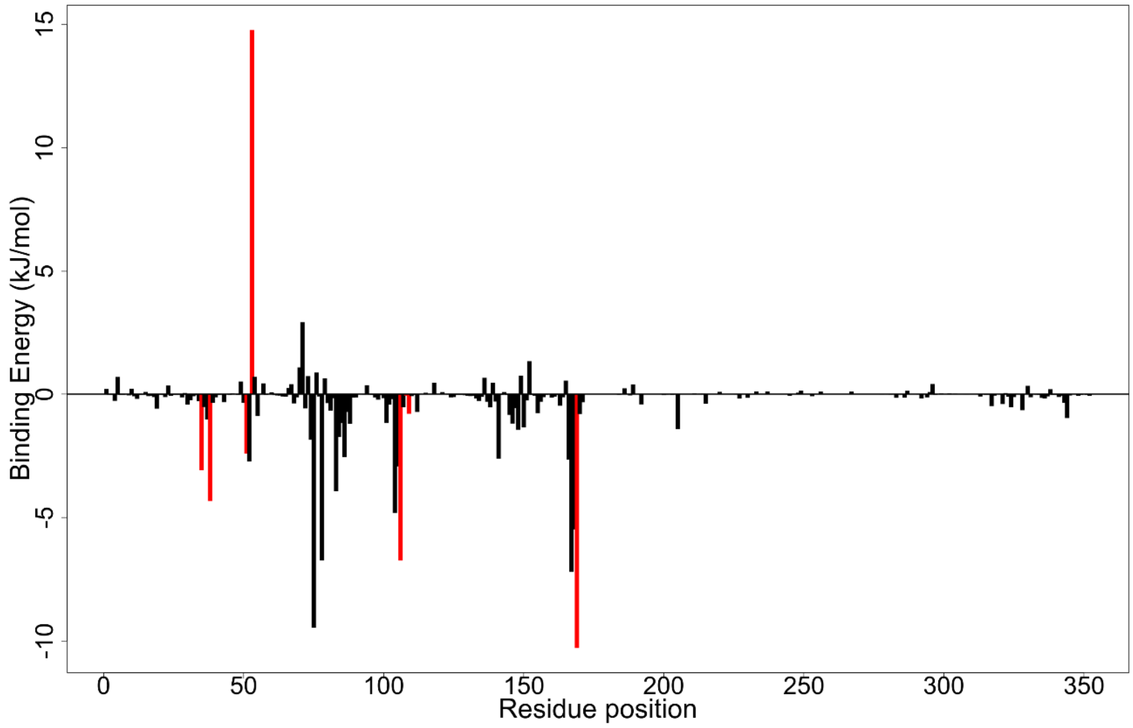

(f)

Figure S4. Molecular mechanics Poisson-Boltzmann surface area (MM-PBSA) plot of binding free energy contribution per-residue for (a) ZINC1691180, (b) ZINC4520996, (c) ZINC95486106, (d) ZINC5733756, (e) SB 202190, and (f) SB 203580-p38 MAPK complexes. Fluctuations of the residues Tyr35, Val38, Ala51, Lys53, Thr106, Leu108, Met109 and Phe169 are coloured red.
